# When is the right time to initiate rehabilitation? Time will tell…

**DOI:** 10.1113/EP091702

**Published:** 2024-03-17

**Authors:** Sarah M. Greising, Jarrod A. Call

**Affiliations:** ^1^ School of Kinesiology University of Minnesota Minneapolis Minnesota USA; ^2^ Department of Physiology and Pharmacology University of Georgia Athens Georgia USA; ^3^ Regenerative Bioscience Center University of Georgia Athens Georgia USA

**Keywords:** injury, skeletal muscle, volumetric muscle loss

## INTRODUCTION

Connections link a sequence of three related research papers. The central article which links the other two papers has been published in *Experimental Physiology*. In a Connections article, an author (or authors) of the central article out lines its principal novel findings, tracing how they were influenced by the first article and how the central article has contributed to the developments made in the third article. The author(s) may also speculate on the direction of future research in the field. Connections articles aim to set the research in a wide context.

When is the right time for anything? Who knows? Living is an art, not a science.

Benjamin Alire Saenz (poet)

Rehabilitation sciences is the field of investigating interventions to facilitate tissue repair towards its pre‐injury function, and similar to life, there often seem to be more uncertainties and unknowns than answers. Nonetheless, the dichotomy of the ‘unknown’ and ‘established theory’ is what lures many to spend our lives in a scientific pursuit. One such pursuit is defining evidence‐based practices for rehabilitation by interrogating injury pathophysiology, testing task‐specific techniques and optimizing rehabilitation programmes in terms of intensity, duration and timing. ‘When is the right time to initiate rehabilitation?’ is not a new question; it is well articulated by the rhetorical question posed by Paul Brown in his historical thoughts on rehabilitation after the Vietnam War: “Leaders and innovators in the field were asking the question— ‘Where does treatment cease and rehabilitation start?’…” (Burkhalter, 1994). Yet, when the optimal time might be to start rehabilitation after various injuries and, in particular, skeletal muscle injuries, remains a looming question.

Skeletal muscle has a robust repair and regenerative capacity after various forms of injury. As a target tissue for rehabilitation, knowing when to start is expected to be a key part to successful recovery. Skeletal muscle is highly responsive to a range of physiological stimuli and cues. For example, progressive aerobic or anaerobic training programmes can take a ‘couch potato’ to ‘5K finisher’ in no time. One key to these programmes is the progressive changes in overload on the system. Successful training programmes usually involve various modalities, types, durations and intensities of exercise. Thus, the programmes are likely to include stretching (or range‐of‐motion) exercises, isometric exercises, dynamic exercises and aerobic exercises. The progressive and phased approach to rehabilitation is no different. Following injuries, the responsiveness of muscle is still there; that is, until it is not. Large‐scale skeletal muscle injuries, such as volumetric muscle loss injuries, are, unfortunately, a great example of the ‘until it is not’. Volumetric muscle loss injures are the traumatic or surgical loss of muscle resulting in irrecoverable impairments in function, and to date, the responsiveness to rehabilitation has been modest to non‐existent.

In the work by Basten et al. (2023), we sought to control the overload of rehabilitation tightly, with a phased approach using both passive range‐of‐motion exercise and isometric muscle training (i.e., neuromuscular electrical stimulation). Using a rodent model of volumetric muscle loss injury to the posterior compartment (soleus, plantaris and gastrocnemius muscles), rehabilitation was initiated early, at 3 days post‐injury, and was structured twice per week for 8 weeks, with a single change in overload at the 4‐week point. In comparison to the field at large, initiating rehabilitation 3 days post‐injury was ‘early’, because most studies have been starting interventions ≥7 days post‐injury. In efforts to understand how activity could impact the functional response to rehabilitation, we compared an experimental group that underwent the same rehabilitation programme but had restricted housing for the duration of the experiment, which could allow for highly targeted interventions. Akin to what others have shown across the volumetric muscle loss field, the rehabilitation was not able to recover the injury‐induced functional impairments, even with the ‘early’ intervention. However, restricting activity was able to mitigate some negative functional impairments, but this was at the expense of impaired whole‐body metabolism.

The task‐specific and early rehabilitation protocol that Basten et al. (2023) designed is common clinically, as exemplified by the collective work of Bayer et al. including the Tendon Research Group Bispebjerg (2017) on muscle strain injuries. Their clinical study directly compared the initiation of rehabilitation at 2 versus 9 days post‐injury, with all participants following the same phased and progressive programme. All injuries were considered ‘structural muscle injuries’ or muscle strains to the lower leg (hamstring, quadriceps or calf muscle complexes), and most participants had a minor to moderate partial muscle tear, with only 1 of 42 participants having a sub‐total muscle tear. The rehabilitation plan was phased through range‐of‐motion exercises, isometric exercises and dynamic exercises. The ‘When is the right time?’ question for the study design was, ‘How soon should range‐of‐motion exercises be started?’. Structurally within the muscle, the extracellular matrix, or the connective tissue of the muscle providing architectural support to the muscle, can be impacted significantly during prolonged limitations on physical activity or immobilization. Thus, for a muscle strain‐type injury, as evaluated by Bayer and colleagues, an early start to range‐of‐motion exercises was proposed to be beneficial. With the primary outcome of ‘successful return to sport’, the early‐intervention group was able to return on average almost 3 weeks earlier than the late‐intervention group. Of note, maximal muscle function following injury was not evaluated, but with return to sport it is implied that function returned. Collectively, the studies by Bayer et al. (2017) and Basten et al. (2023), both involving range‐of‐motion exercise, beg the question, ‘Should the plasticity of the extracellular matrix be guiding the decision on when to initiation rehabilitation?’. This question has been overlooked by the field to date. Mechanistically, it is important to highlight that injuries such as muscle strain leave the extracellular matrix intact, whereas in injuries such as volumetric muscle loss injury, the extracellular matrix is completely lost from the injured area.

**Connected Articles**

- Bayer ML, Magnusson SP, Kjaer M; Tendon Research Group Bispebjerg. Early versus delayed rehabilitation after acute muscle injury. *N Engl J Med*. 2017;377(13):1300–1301.
- Basten AM, Raymond‐Pope CJ, Hoffman DB, Call JA, Greising SM. Early initiation of electrical stimulation paired with range of motion after a volumetric muscle loss injury does not benefit muscle function. *Exp Physiol*. 2023;108(1):76–89.
- Motherwell JM, Dolan CP, Kanovka SS, Edwards JB, Franco SR, Janakiram NB, Valerio MS, Goldman SM, Dearth CL. Effects of adjunct antifibrotic treatment within a regenerative rehabilitation paradigm for volumetric muscle loss. *Int J Mol Sci*. 2023;24(4):3564.

More recently, Motherwell et al. (2023), took a delayed approach to rehabilitation following volumetric muscle loss injury, with a start 7 days post‐injury. This preclinical study used voluntary wheel running as a rehabilitation‐like intervention for ∼7 weeks. Interventions such as voluntary wheel running rely on unplanned bouts that can vary in duration and intensity across animals. However, most of the animals in the work by Motherwell did run on average 1–3 km/day, which exceeds a threshold for known positive adaptations to muscle. Motherwell also adopted a regenerative rehabilitation approach, which combined rehabilitation with an antifibrotic pharmaceutical and skeletal muscle graft (i.e., minced graft that delivers all necessary aspects: muscle stem cells, satellite cells and the endogenous extracellular matrix). Collectively, the greatest functional improvements were found when the intervention did not include the antifibrotic treatment. The antifibrotic pharmaceutical used (losartan) acts by inhibiting angiotensin II type 1 receptor activation and thus blocks transforming growth factor‐β1 and connective tissue growth factor activity, which might negatively impact the endogenous muscle extracellular matrix and thus limit stabilization of the injured muscle when rehabilitation is combined. The extracellular matrix has a partial fibrotic tissue component and there are two key elements of the extracellular matrix that are important to consider: first, the structural support that it lends to the muscle; and second, that pathological overproduction of extracellular matrix that can drive development of fibrosis in the muscle after injury. Although rehabilitation‐based running did not improve muscle function alone, it is notable that the minced muscle graft (without additional antifibrotic treatment) did support improvements. Thus, taking into consideration the plasticity and stability of the extracellular matrix, both the endogenous and exogenous components, while deciding when to start rehabilitation might be a new avenue for the field.

So yes, ‘living [may be] an art, not a science’, but there is hope that rehabilitation can be rooted in evidenced‐based practices supported by strong preclinical investigations. The connections made across the three studies exemplify the challenges of identifying the appropriate time to initiate rehabilitation, but elicit exciting new questions with respect to the role of the extracellular matrix in guiding rehabilitation decisions. We look forward to creative investigations and cross‐disciplinary partnerships evaluating both the endogenous and exogenous extracellular matrix throughout rehabilitation and recovery.

## AUTHOR CONTRIBUTIONS

Sarah M. Greising and Jarrod A. Call approved the final version of the manuscript and agree to be accountable for all aspects of the work. All persons designated as authors qualify for authorship, and all those who qualify for authorship are listed.

## CONFLICT OF INTEREST

None declared.
